# Multiplexed analysis of macrophage polarisation in pulmonary metastases of microsatellite stable colorectal cancer

**DOI:** 10.1007/s00262-024-03646-0

**Published:** 2024-02-22

**Authors:** Topias Karjula, Hanna Elomaa, Sara A. Väyrynen, Teijo Kuopio, Maarit Ahtiainen, Olli Mustonen, Iiris Puro, Anne Niskakangas, Jukka-Pekka Mecklin, Jan Böhm, Erkki-Ville Wirta, Toni T. Seppälä, Eero Sihvo, Fredrik Yannopoulos, Olli Helminen, Juha P. Väyrynen

**Affiliations:** 1 Translational Medicine Research Unit, Medical Research Center Oulu, Oulu University Hospital and University of Oulu, Oulu, Finland; 2 Department of Biological and Environmental Science, University of Jyväskylä, 40014 Jyväskylä, Finland; 3 Department of Education and Research, Hospital Nova of Central Finland, Well Being Services County of Central Finland, 40620 Jyväskylä, Finland; 4 Department of Internal Medicine, Oulu University Hospital, Oulu, Finland; 5 Department of Pathology, Hospital Nova of Central Finland, Well Being Services County of Central Finland, 40620 Jyväskylä, Finland; 6 Faculty of Sport and Health Sciences, University of Jyväskylä, 40014 Jyväskylä, Finland; 7 Faculty of Medicine and Health Technology, Tampere University and TAYS Cancer Center, Tampere University Hospital, 33520 Tampere, Finland; 8 Department of Gastrointestinal Surgery, Helsinki University Central Hospital, University of Helsinki, 00290 Helsinki, Finland; 9 Applied Tumor Genomics, Research Program Unit, University of Helsinki, 00290 Helsinki, Finland; 10 Department of Gastroenterology and Alimentary Tract Surgery, Tampere University Hospital and TAYS Cancer Centre, 33520 Tampere, Finland; 11 Central Hospital of Central Finland, 40014 Jyväskylä, Finland; 12 Department of Cardiothoracic Surgery, Oulu University Hospital, Oulu, Finland

**Keywords:** Pulmonary metastases, Colorectal cancer, Tumour-associated macrophages, Macrophage polarisation, Cancer immunology

## Abstract

Tumour-associated macrophages (TAMs) express a continuum of phenotypes ranging from an anti-tumoural M1-like phenotype to a pro-tumoural M2-like phenotype. During cancer progression, TAMs may shift to a more M2-like polarisation state, but the role of TAMs in CRC metastases is unclear. We conducted a comprehensive spatial and prognostic analysis of TAMs in CRC pulmonary metastases and corresponding primary tumours using multiplexed immunohistochemistry and machine learning-based image analysis. We obtained data from 106 resected pulmonary metastases and 74 corresponding primary tumours. TAMs in the resected pulmonary metastases were located closer to the cancer cells and presented a more M2-like polarised state in comparison to the primary tumours. Higher stromal M2-like macrophage densities in the invasive margin of pulmonary metastases were associated with worse 5-year overall survival (HR 3.19, 95% CI 1.35–7.55, *p* = 0.008). The results of this study highlight the value of multiplexed analysis of macrophage polarisation in cancer metastases and might have clinical implications in future cancer therapy.

**Supplementary Information:**

The online version contains supplementary material available at 10.1007/s00262-024-03646-0.

## Introduction

Tumour-associated macrophages (TAMs) are innate immune cells differentiated from extravasated monocytes and tissue-resident macrophages at the cancer site. They represent a major immune cell population in the tumour microenvironment (TME) [1] and have remarkable plasticity in the regulation of tissue homeostasis and inflammation [2]. Macrophage polarisation refers to the activation state of macrophages at a singular point in time [2]. TAMs express a continuum of phenotypes ranging from an anti-tumoural M1 to a pro-tumoural M2 at the extremes. Apart from affecting tumour growth and prognosis, TAMs are known to be essential players in cancer therapy partly mediating the impact of pre-operative treatments [3, 4], but also causing chemotherapy resistance [5].

Colorectal cancer (CRC) is one of the leading causes of cancer mortality globally [6]. The prognostic value of macrophages in CRC has been evaluated by several studies [7, 8], and a recent meta-analysis concluded that higher overall TAM density is associated with a favourable prognosis, the association being more prominent in mismatch repair (MMR) proficient CRC patients [7]. Higher densities of M2-like macrophages are shown to be associated with worse prognosis and disease recurrence [8–10] and TAMs have been proposed to shift towards an M2 polarisation state alongside disease progression [9, 11]. There are also contradictory reports on the prognostic effect of TAMs in CRC [12, 13]. Challenges in TAM research reproducibility may arise from the use of single macrophage markers, varying density assessment methods, and potential confounding factors such as cancer subtypes, anatomical location, and TAM subtype distribution within tumours [14, 15].

The role of the immune system in metastatic dissemination is a growing field in cancer research. Recent advances in cancer immunotherapy targeting TAMs excite promises in treating advanced stages of cancer [16]. However, research on the significance of TAMs in CRC metastases is limited. Approximately 5–10% of CRC patients have synchronous pulmonary metastases and around 5% develop disease recurrence with pulmonary metastases within 5 years of primary tumour treatment [17, 18]. While the 5-year overall survival of CRC across all stages exceeds 60%, patients with stage IV CRC have a 5 year survival of only 14% [19]. In metastatic dissemination, circulating monocytes and tissue-resident macrophages are proposed to have a major contribution in the formation of premetastatic niches preceding metastasis formation [16]. In surgically operated CRC liver metastases, higher densities of M2-like macrophages are associated with shorter disease recurrence [20]. Also, the larger size of M2-like TAMs has been linked with worse survival [21]. However, the prognostic value of TAMs in CRC pulmonary metastases remains unexplored.

In this study, we conducted a comprehensive spatial and prognostic analysis of the TAMs in resected pulmonary metastases of CRC with a comparison to corresponding primary tumours. We used multiplex immunohistochemistry and machine learning-based image analysis that enabled the recognition of single TAMs and their phenotyping based on the expression of multiple polarisation markers.

## Material and methods

### Study design

This retrospective, population-based study included all patients with histologically confirmed pulmonary metastases from CRC operated in Oulu University Hospital and Central Finland Central Hospital during 2000–2020. The study hospitals serve as the sole providers of thoracic surgery in their districts. In total, 106 pulmonary metastasectomies from CRC were performed on 74 patients during the study period at these hospitals. The eligibility for pulmonary metastasectomy was determined based on the potential for surgical resection to provide curative treatment.

### Data collection

Patients were identified by reviewing surgical registries and pathology reports. Relevant clinical data were retrospectively collected from electronic patient record systems. The tumour classification was updated to the 8th edition of the Union for International Cancer Control (UICC) tumour-node-metastasis (TNM) classification [22]. Survival data until December 31, 2021 were obtained from Statistics Finland, with the follow-up data being 100% complete. Haematoxylin and eosin (H&E) stained slides of the primary tumours and pulmonary metastases were retrieved from pathology archives and reviewed by a pathologist. The most representative slide of the primary tumours showing the deepest invasion depth was chosen for further analysis. In patients with multiple metastases resected at first pulmonary metastasectomy, the representative histological sample was chosen arbitrarily. The slides were digitalised using a NanoZoomer XR (Hamamatsu Photonics, Hamamatsu City, Japan) or Aperio AT2 (Leica Biosystems Imaging Inc., Wetzlar, Germany) scanner equipped with a 20 × objective.

### (23)Tissue microarray

**Fig. 1:**
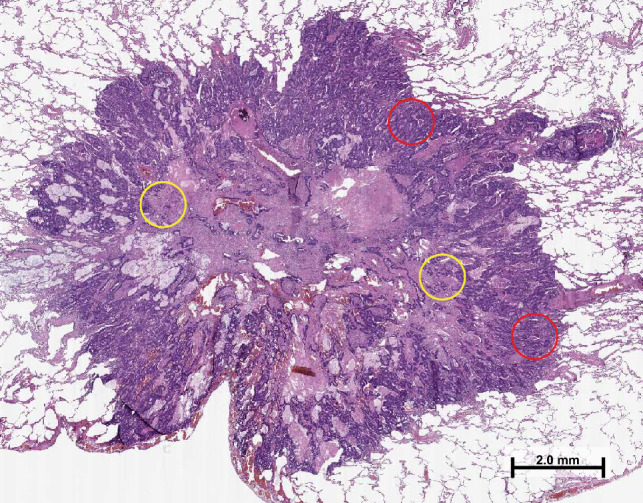
A haematoxylin–eosin-stained section of a pulmonary metastasis illustrating the selection of tissue microarray core locations. The red circles indicate tumour cores from the invasive margin and yellow circles indicate tumour cores from the tumour centre

For tissue analyses, tissue microarray (TMA) blocks were prepared from formalin-fixed paraffin-embedded tissue samples using a 1-mm core diameter. We obtained 2 cores from the tumour centre and 2 cores from the invasive margin from both the metastasis and the primary tumour. The precise core locations were determined from the digitalised H&E-stained slides and were chosen to best represent overall tumour morphology while avoiding necrosis. Figure 1 illustrates core location selection for tissue microarrays. The invasive margin tumour cores (red circles) were picked so that they included tumour epithelium and peritumoural healthy tissue. Tissue microarray cores from the tumour centre (yellow circles) were obtained more centrally. The TMA blocks were cut into a 3.5 µm-thick sections for further staining and analysis. The samples were screened for DNA mismatch repair deficiency with MLH1, MSH2, MSH6, and PMS2 immunohistochemistry and *BRAF* V600E mutation status with mutation-specific immunohistochemistry (clone VE1), as described previously [23]. All patients were MMR proficient.

### Multiplex immunohistochemistry

We designed a 9-plex immunohistochemistry panel [24], of which 6 markers were used in this analysis: Macrophages were identified using CD68 marker, and further phenotyping to M1 and M2 polarisation was performed with 4 markers [M1: HLADR and CD86; M2: CD163 and MRC1(CD206)]. Additionally, keratin (KRT) was used to detect tumour cells. We used the standardised nomenclature system for genes and gene products (www.genenames.org) to improve clarity and reduce ambiguity as recommended by the expert panel [25]. The multiplex immunohistochemistry staining was done with Bond-III automated IHC stainer (Leica Biosystems), Bond Refine Detection kit (DS9800, Leica Biosystems), and Dako AEC + High Sensitivity Substrate Chromogen (K3469, Agilent, Santa Clara, CA, USA) as previously described [24]. The procedure included staining one marker at a time, scanning the slide, heat-mediated antibody removal, and AEC removal with ethanol [24]. Candidate antibodies and suitable dilutions were optimised in a test tissue microarray consisting of normal colorectal mucosa, colorectal cancer tissue and tonsil tissue. These antibodies were then combined into a multiplex immunohistochemistry assay that was validated by confirming similar staining patterns of multiplex and conventional immunohistochemistry [24]. All slides were stained in one batch.

### Image analysis

**Fig. 2:**
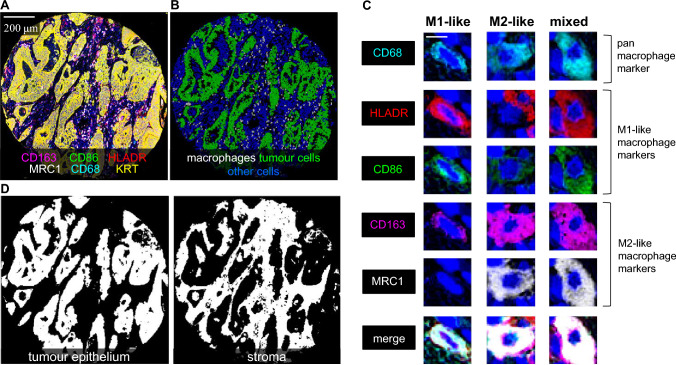
Multiplex immunohistochemistry visualisation of macrophage phenotypes and image analysis. **A**, 6-plex immunohistochemistry image, in which each marker is represented with a unique colour. **B**, Detection and phenotyping cells into macrophages, tumour cells, and other cells in QuPath bioimage software using machine-learning based algorithms. **C**, Examples of three macrophages with an M1-like, M2-like, and mixed-like polarisation phenotype. **D**, The segmentation of tissue compartments into tumour epithelium and stroma. The length of the scale bar is 2.5 µm

The images of multiplex immunohistochemistry slides were processed using QuPath [26]. Tissue microarray cores were recognised with the *TMA dearrayer* function and separated into single-core images. We excluded images of cores which were folded or detached during processing, included a minimal amount of tumour, or were necrotic. The single core images of all staining cycles were merged into a pseudo-coloured multiplex immunohistochemistry image (pseudo-immunofluorescence image) using Fiji ImageJ open-source software [27] (Fig. 2A). The haematoxylin channel was used for aligning cell nuclei [24]. The cells were detected and phenotyped into macrophages, tumour cells, and other cells using QuPath-based machine-learning algorithms (Fig. 2B) [24]. The staining pattern for each marker in three example cells is shown in Fig. 2C.

The pseudo-immunofluorescence images were further processed in QuPath, where cells and tissue compartments were detected and phenotyped using previously validated supervised machine learning algorithms [28]. The cells were identified with the *cell detection* function and phenotyped into macrophages, tumour cells, and other cells (Fig. 2B) as described previously [24]. For tissue categorisation, QuPath was trained to identify segments of tumour epithelium and stroma (Fig. 2D) using the built-in *pixel classifier* function [24].

### Scoring and classification

Macrophages were classified according to their M1/M2 polarisation state. To calculate a polarisation index for each macrophage, staining intensities of the four macrophage markers were first converted into percentile scores across all macrophages. Then, a polarisation index for each macrophage was calculated by subtracting the percentile scores of M2-like macrophage markers from the M1-like macrophage markers [formula: (CD86 + HLA-DR)—(CD163 + MRC1); with marker names denoting intensity percentile scores]. The index values were then divided into quartile categories (Q1–Q4) across all pulmonary metastases and primary tumours. Following the previous study [24], macrophages in the lowest quartile were classified as M1-like macrophages and those in the highest quartile as M2-like macrophages. Macrophages in the middle quartiles (Q2–Q3) were classified as mixed-type macrophages and excluded from further analysis. The average densities of M1- and M2-like macrophage subsets in tumour epithelial and stromal compartments of the tumour centre and invasive margin were calculated for each case. Survival analysis for each subset was performed with a two-tiered classification (low/high) using macrophage density cutoffs picked from the receiver-operating characteristics (ROC) curve (Table S1). Including macrophages in all tissue compartments and tumour regions (epithelial and stromal areas in both the invasive margin and tumour centre), an integrative macrophage polarisation score (M1:M2 density ratio) was calculated to evaluate the prognostic value of M1:M2 polarisation in the whole tumour. The cutoffs selected based on ROC analysis are presented in Table S1.

Intratumoural heterogeneity of M1-like and M2-like macrophage densities were analysed using the standard deviations (SDs) of macrophage densities in all tumour cores. The SD values were separately calculated for tumour epithelial and stromal regions, and those were only analysed from tumours with more than one representative tumour core. Using cutoffs selected based on ROC analysis, the prognostic effect of intratumoural heterogeneity (low/high SD) of macrophage densities was analysed.

To analyse spatial interactions between macrophages and tumour cells, we used *R* programming language (version 4.0.3) and the *spatstat* (2.2–0) package to calculate nearest neighbour distances (NNDs) from macrophages to their closest neighbour points of specific categories (e.g. tumour cells). To visualise the results, scaled intensities of macrophage polarisation markers were plotted against NNDs from tumour cells using the *ggplot2* (3.3.3) package and generalised additive model smoothing [formula *y* ~ *s*(*x*)].

### Outcomes and definitions

Royal College of Surgeons Charlson Score (RCSCS) was employed for comorbidity classification [29]. The cancer under treatment was also considered as one of the comorbidities. The disease-free interval (DFI) was defined as the time between primary tumour surgery and the detection of first pulmonary metastases. Pulmonary metastases found within 6 months after primary cancer treatment were categorised as synchronous, while those detected later as metachronous. The primary outcome of the study was 5-year overall survival from pulmonary metastasectomy to death due to any cause before the end of follow-up. Only one patient died from a cause other than cancer and, therefore, cancer-specific survival was not analysed.

### Statistical analysis

For group comparison of categorical variables, the Chi-square test and Fisher’s exact test were employed. Continuous variable group comparisons were conducted using the Student’s *T* test and Mann–Whitney *U* test. Bivariate correlation analysis was performed using Spearman correlation coefficients. To visualise survival up to 5 years after pulmonary metastasectomy, Kaplan–Meier survival curves were constructed from the first metastasectomy to death or end of follow-up. The estimates for hazard ratios (HR) along with their 95% confidence intervals (CI) were calculated using Cox proportional hazards regression. The multivariable models were adjusted for sex (male/female), age (continuous variable), comorbidity (RCSCS 1/ ≥ 2), neoadjuvant therapy (no/yes), number of pulmonary metastases at diagnosis (1/ ≥ 2), former liver metastases (no/yes), and synchronicity of pulmonary metastases (synchronous/metachronous). The selection of covariates was based on the prior literature [30–32]. Hazard ratios for covariates for 5-year overall survival are presented in Supplementary Table S2. Patients receiving an incomplete R1 resection of the metastases and patients deceased during 30 post-operative days were excluded from the survival analysis. Statistical analysis was performed using IBM SPSS Version 28 (IBM Corp., Armonk, NY, USA), Rstudio (version 1.3.1093), and *R* statistical programming (version 4.0.3).

### Ethical aspects

The Oulu University Hospital Ethics Committee (EETMK 81/2008) approved the study. The Finnish National Authority of Medicolegal Affairs (VALVIRA) waived the need for informed consent due to the retrospective nature of the study (D.no 3916/06.01.03.01/2016). The study was performed in accordance with the Declaration of Helsinki.

## Results

### Baseline characteristics

During the study period, a total of 106 pulmonary metastasectomies were performed on 74 patients. Among the metastasectomies, 36 cases were re-metastasectomies conducted on 21 patients. The median DFI after primary CRC surgery was 337 (IQR 0–783) days. Of the patients, 12 (16%) had bilateral pulmonary metastases and 25 (34%) had more than one pulmonary metastasis. In 4 patients (5.7%), an R1 resection of pulmonary metastases was recorded. Of the patients, 32 (43%) had been diagnosed and previously treated for liver metastasis of CRC. The median follow-up time was 26.5 months (IQR 18.6–48.48, range 1–209 months). The overall 5-year survival rate was 32%.

### Macrophage densities and polarisation

We successfully analysed 291 TMA cores from 91 CRC pulmonary metastases and 153 TMA cores from 54 primary tumours. The median macrophage density in pulmonary metastases was 712 cells/mm^2^ in the invasive margin and 485 cells/mm^2^ in the tumour centre. The overall CD68^+^ macrophage densities were higher in the first resected metastases compared to the re-metastasectomies; however, the difference was statistically significant only in the epithelial and overall compartments in the tumour centre (Supplementary Table S3). In the primary tumours, the median macrophage densities in the invasive margin and tumour centre were 588 cells/mm^2^ and 425 cells/mm^2^, respectively.

**Fig. 3:**
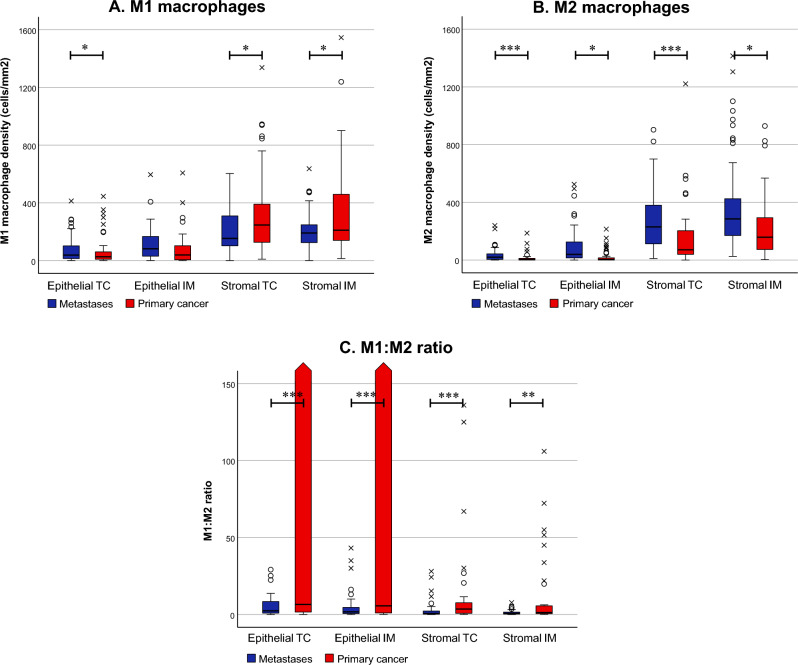
Macrophage polarisation in primary colorectal tumours and the corresponding first resected pulmonary metastases. Comparison of M1- (**A**) and M2-like macrophage densities (**B**) and M1:M2 ratios (**C**) between the primary tumours and pulmonary metastases. Rounds indicate outliers, and crosses indicate extreme outliers. The third and fourth quartile values for epithelial M1:M2 ratios were 203,503,770 and 20,083,912,500 in the tumour centre (TC) and 692,847,301 and 11,470,838,740 in the invasive margin of the primary tumours, respectively. ***p < 0.001, **p < 0.01, *p < 0.05. The statistical significance was tested with the Wilcoxon signed-rank test. Strom = stromal

The distribution of M1- and M2-like macrophage densities in the first resected pulmonary metastases is presented in Fig. 3. Macrophages were mainly distributed in the stromal areas of the metastases, and within the stromal/epithelial compartments, the densities did not vary between the biopsy core location of the tumour (Supplementary figures S1 and S2). M1- and M2-like macrophage densities in the epithelial compartment had a positive correlation with each other, whereas in the stromal compartment, the correlation was inverse (Table S4). The median M1:M2 density ratio in the invasive margin of the first resected pulmonary metastasis was 1.7 in the epithelial and 0.6 in the stromal compartment. There was no difference in the M1:M2 density ratios between the first resected pulmonary metastasis and re-metastasectomies (Table S3).

In comparison with the primary tumours, the pulmonary metastases presented a more pro-tumoural macrophage polarisation with significantly higher M2-like macrophage densities, lower stromal M1-like macrophage densities, and significantly lower M1:M2 density ratios (Fig. 3). In the correlation analysis between first resected pulmonary metastases and primary tumours, only epithelial M1-like macrophage densities in the primary tumour centre had a statistically significant correlation with the epithelial M1-like and M2-like macrophage densities in the tumour centre of the first pulmonary metastasis (Table S5).

### Intratumoural heterogeneity of macrophage densities

The medians of standard deviations for epithelial M1-like and M2-like macrophage densities, and stromal M1-like and M2-like macrophage densities in different tumour cores of the first resected pulmonary metastases were 57, 24, 87, and 99 cells/mm2, respectively. Whereas in primary tumours, the medians of standard deviations for epithelial M1-like and M2-like macrophage densities and stromal M1-like and M2-like macrophage densities in different tumour cores were 23, 5, 115, and 93 cells/mm2, respectively. Using standard deviations as a measure of intratumoural density heterogeneity, only stromal M2-like macrophages in the pulmonary metastases had higher heterogeneity compared to the primary tumours (*p* < 0.001).

### Macrophage spatial analysis

**Fig. 4:**
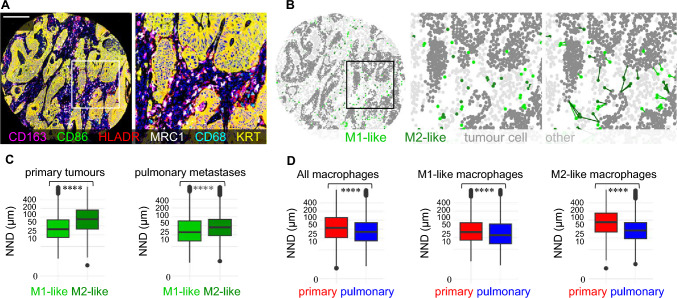
Spatial analysis of macrophages in primary tumours and in pulmonary metastases using the nearest neighbour distance (NND) function. **A**, Pseudocoloured multiplex immunohistochemistry image from an example tissue microarray core and from a smaller tumour region. **B**, Cell phenotyping maps with nearest neighbour distance analysis from each M1-like and M2-like macrophage to the closest tumour cell. **C**, Boxplots visualising the nearest neighbour distances (NNDs) from M1-like macrophages and M2-like macrophages to the closest tumour cell across all tumour images of primary tumours and pulmonary metastases. **D**, Boxplots visualise the distribution of nearest neighbour distances from all macrophages, M1-like macrophages, and M2-like macrophages to the closest tumour cell in primary tumours and pulmonary metastases. The results are based on 27,775 M1-like and 39,386 M2-like macrophages in the pulmonary metastases, and 146,438 M1-like and 83,159 M2-like macrophages in the primary tumours. The statistical significance was tested with the Wilcoxon rank-sum test. ****P < 0.0001

The median NNDs from macrophages to tumour cells in the first resected pulmonary metastases were 31 µm for M1-like and 45 µm for M2-like macrophages; M1-like macrophages were 32% closer to the tumour cells compared to M2 macrophages. In primary tumours, M1-like macrophages were 48% closer to the tumour cells than M2-like macrophages. The macrophages were also closer to the tumour cells in pulmonary metastases compared to primary tumours (Fig. 4).

### Macrophage polarisation and survival

**Fig. 5:**
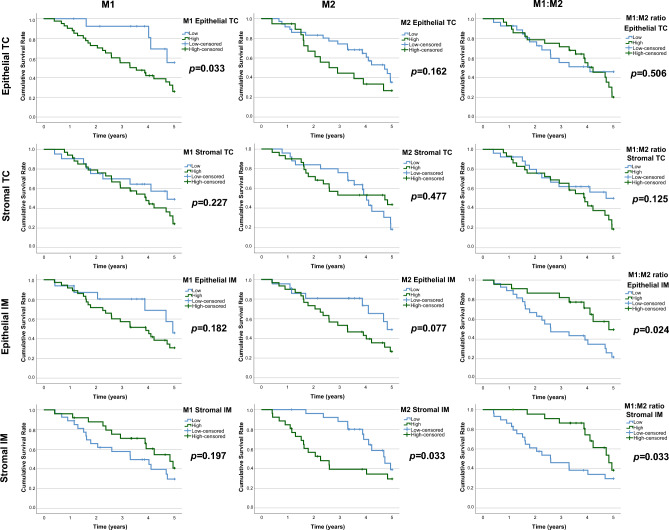
Kaplan–Meier survival analysis of macrophage densities. The plots present 5-year overall survival curves stratified by epithelial and stromal M1-like and M2-like macrophage densities and M1:M2 ratios in the tumour centre (TC) and the invasive margin (IM) of the first resected pulmonary metastases of CRC. Log-rank tests were applied

**Fig. 6:**
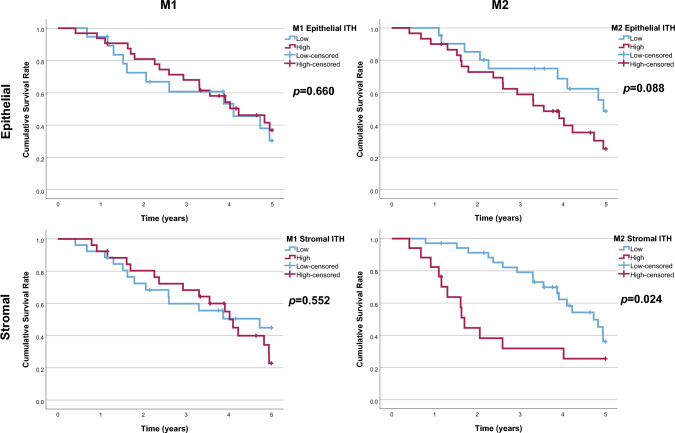
Kaplan–Meier survival analysis of intratumoural heterogeneity (ITH) of macrophage infiltrates. The plots present 5-year overall survival curves stratified by low versus high standard deviations of M1-like and M2-like macrophage densities in the tumour epithelial and stromal compartments of the first resected pulmonary metastases. Log-rank tests were applied

When macrophages were classified according to their polarisation, high M2-like macrophage density in the invasive margin of pulmonary metastases was associated with worse 5-year overall survival (stromal: low 39% vs. high 30%, *p* = 0.033, epithelial: low 49% vs. high 27%, *p* = 0.077; Fig. 5). High epithelial M1-like macrophage densities in the tumour centre of the pulmonary metastases were associated with worse survival. High M1:M2 ratios in the invasive margins of the pulmonary metastases were associated with longer 5-year overall survival (epithelial: low 22% vs. high 50%, *p* = 0.024; stromal: low 30% vs. high 38%, *p* = 0.033; Fig. 5). In Cox multivariable analysis, high stromal M2 macrophage densities in the invasive margin of the pulmonary metastases were significantly associated with worse survival (adjusted HR 3.19, 95% CI 1.35–7.55, *p* = 0.008; Table 1). High epithelial M1-like macrophage densities in the tumour centre of the pulmonary metastases were also associated with worse survival (adjusted HR 3.58, 95% CI 1.13–11.41, *p* = 0.031; Table 1). Furthermore, high M1:M2 density ratios (a more M1-like macrophage polarisation) in the invasive margin of the pulmonary metastases were significantly associated with favourable prognosis in both stromal (adjusted HR 0.17, 95% CI 0.07–0.46, *p* < 0.001; Table 1) and epithelial compartments (adjusted HR 0.23, 95% CI 0.09–0.59, *p* = 0.002; Table 1). The integrative macrophage polarisation score, combining macrophage densities of all regions in the tumours, did not have a significant effect of 5-year overall survival in univariate (low 42.0% vs. high 28.2%; *p* = 0.699; Figure S3) or multivariate analysis (high vs. low score: adjusted HR 0.672; 95% CI 0.33–1.35; *p* = 0.263). When analysing the prognostic effect of intratumoural heterogeneity of macrophage densities separately from the stromal and epithelial compartments, high intratumoural M2-like macrophage density heterogeneity was significantly associated with worse 5-year overall survival (low 36.1% vs. high 25.5%, *p* = 0.024; Fig. 6).

**Table 1:** Hazard ratios with 95% confidence intervals for 5 year all-cause mortality according to M1- and M2-like macrophage densities in the first pulmonary metastases

	M1-like macrophage density		M2-like macrophage density		M1:M2 ratio	
	Low, HR (95%CI)	High, HR (95%CI)	Low, HR (95%CI)	High, HR (95%CI)	Low, HR (95%CI)	High, HR (95%CI)
Tumour centre						
Stomal						
Crude	1.00 (reference)	1.44 (0.71–2.93; *p* = 0.314)	1.00 (reference)	0.87 (0.45–1.69; *p* = 0.679)	1.00 (reference)	1.48 (0.76–2.91; *p* = 0.250)
Adjusted*	1.00 (reference)	1.70 (0.70–4.09; *p* = 0.243)	1.00 (reference)	0.70 (0.33–1.50; *p* = 0.359)	1.00 (reference)	1.69 (0.79–3.62; *p* = 0.180)
Epithelial						
Crude	1.00 (reference)	2.20 (0.91–5.28; *p* = 0.079)	1.00 (reference)	1.67 (0.86–3.24; *p* = 0.128)	1.00 (reference)	1.22 (0.63–2.38; *p* = 0.558)
Adjusted*	1.00 (reference)	3.58 (1.13–11.41; ***p* = 0.031**)	1.00 (reference)	2.0 (0.86–4.63; *p* = 0.109)	1.00 (reference)	1.03 (0.48–2.22; *p* = 0.940)
Invasive margin						
Stromal						
Crude	1.00 (reference)	0.62 (0.30–1.25; *p* = 0.181)	1.00 (reference)	2.17 (1.06–4.42; ***p* = 0.033**)	1.00 (reference)	0.41 (0.19–0.87; ***p* = 0.020**)
Adjusted*	1.00 (reference)	0.49 (0.21–1.15; *p* = 0.102)	1.00 (reference)	3.19 (1.35–7.55; ***p* = 0.008**)	1.00 (reference)	0.17 (0.07–0.46; ***p* < 0.001**)
Epithelial						
Crude	1.00 (reference)	1.55 (0.69–3.44; *p* = 0.287)	1.00 (reference)	1.72 (0.81–3.64; *p* = 0.156)	1.00 (reference)	0.44 (0.21–0.93; ***p* = 0.031**)
Adjusted*	1.00 (reference)	1.72 (0.64–4.58; *p* = 0.280)	1.00 (reference)	1.74 (0.68–4.49; *p* = 0.250)	1.00 (reference)	0.23 (0.09–0.59; ***p* = 0.002**)

*Cox proportional hazards regression models adjusted for gender (female/male), age (continuous), RCSCS (1/2/ ≥ 3), neoadjuvant therapy (no/yes), synchronicity of pulmonary metastases (synchronous/metachronous), number of pulmonary metastases at diagnosis (1/ ≥ 1) former liver metastases (no/yes)

Since neoadjuvant therapy is known to affect macrophage polarisation, at least in primary tumours, sensitivity analysis was performed on patients who had not received neoadjuvant therapy. In the sensitivity analysis, M1-like and M2-like macrophage densities or M1:M2 density ratios in the first resected pulmonary metastases were not statistically significantly associated with overall survival; however, there was some trend of a similar survival effect as in the analysis including all patients (Figure S4). In multivariable analysis, high stromal M1:M2 density ratios in the invasive margin was statistically significantly associated with favourable survival (adjusted HR 0.22, 95% CI 0.06–0.89, *p* = 0.035; Table S6).

### Association with clinical parameters

The M1-like and M2-like macrophage densities or M1:M2 ratios in the invasive margin of the pulmonary metastases were not associated with patient characteristics (Table 2, Table S7, and Table S8). In primary tumours, high epithelial M1 macrophage density in the invasive margin of the tumour was associated with lower stage, rectal origin, and neoadjuvant chemotherapy (Table S9). Neoadjuvant chemotherapy in the primary tumours was associated with a shift of the macrophage polarisation towards a more anti-tumoural state; however, the association being statistically significant only in the epithelial M1-like macrophage densities in the invasive margin. In the first resected pulmonary metastases, neoadjuvant chemotherapy was not associated with macrophage densities or polarisation (Table S10).

**Table 2:** Baseline characteristics according to stromal M1- and M2-like macrophage densities in the invasive margin of first resected CRC pulmonary metastases

	M1-like macrophages			M2-like macrophages		*p*
	Low	High		Low	High	
	*n* (%)	*n* (%)		*n* (%)	*n* (%)	
n	30	26		27	29	
Sex			> 0.999			0.789
Female	15 (50.0%)	13 (50.0%)		14 (51.9%)	14 (48.3%)	
Male	15 (50.0%)	13 (50.0%)		13 (48.1%)	15 (51.7%)	
Age (M; SD)	68.27 (11.92)	66.42 (9.16)	0.524	66.93 (10.62)	67.86 (10.88)	0.746
RCS Comorbidity Score			0.617			0.548
1	20 (66.7%)	14 (53.8%)		15 (55.6%)	19 (65.5%)	
2	6 (20.0%)	7 (26.9%)		8 (29.6%)	5 (17.2%)	
≥ 3	4 (13.3%)	5 (19.2%)		4 (14.8%)	5 (17.2%)	
Neoadjuvant chemotherapy			0.666			0.446
No	19 (63.3%)	15 (57.7%)		15 (55.6%)	19 (65.5%)	
Yes	11 (36.7%)	11 (42.3%)		12 (44.4%)	10 (34.5%)	
Disease stage			0.184			0.735
1–2	10 (33.3%)	11 (42.3%)		9 (33.3%)	12 (41.4%)	
3	15 (50.0%)	7 (26.9%)		12 (44.4%)	10 (34.5%)	
4	5 (16.7%)	8 (30.8%)		6 (22.2%)	7 (24.1%)	
Primary tumour location			0.284			0.789
Colon	17 (56.7%)	11 (42.3%)		14 (51.9%)	14 (48.3%)	
Rectum	13 (43.3%)	15 (57.7%)		13 (48.1%)	15 (51.7%)	
Disease-free interval (d; MD; IQR)	675 (143–925)	282.5 (0–763)	0.284	427 (0–1004)	314 (0–773)	0.496
Size of largest pulmonary metastasis (cm; MD; IQR)	2.2 (1.2–3.5)	2.5 (1.5–3.5)	0.424	2 (1.2–3.5)	2.5 (1.4–3.5)	0.938
Former liver metastases			0.197			0.977
No	19 (63.3%)	12 (46.2%)		15 (55.6%)	16 (55.2%)	
Yes	11 (36.7%)	14 (53.8%)		12 (44.4%)	13 (44.8%)	
Synchronicity			0.351			0.889
Synchronous	5 (16.7%)	7 (26.9%)		6 (22.2%)	6 (20.7%)	
Metachronous	25 (83.3%)	19 (73.1%)		21 (77.8%)	23 (79.3%)	
No. of pulmonary metastases			0.337			0.719
1	16 (53.3%)	18 (69.2%)		15 (55.6%)	19 (65.5%)	
2	10 (33.3%)	7 (26.9%)		9 (33.3%)	8 (27.6%)	
≥ 3	4 (13.3%)	1 (3.8%)		3 (11.1%)	2 (6.9%)	
Laterality of metastases			0.093			0.889
Unilateral	21 (70.0%)	23 (88.5%)		21 (77.8%)	23 (79.3%)	
Bilateral	9 (30.0%)	3 (11.5%)		6 (22.2%)	6 (20.7%)	
BRAF			0.935			0.113
Wild-type	27 (96.4%)	24 (96.0%)		22 (91.7%)	29 (100.0%)	
Mutant	1 (3.6%)	1 (4.0%)		2 (8.3%)	0 (0.0%)	

*RCS* royal college of surgeons. The Chi-square test, Student’s *T* test, and Mann–Whitney *U* test were applied

## Discussion

We utilised multiplex immunohistochemistry to perform a detailed analysis of differentially polarised TAMs in the resected pulmonary metastases of CRC, with a comparison to the primary tumours. The main finding is that TAMs express a more pro-tumoural M2-like phenotype in the pulmonary metastases in comparison to the corresponding primary tumours, and the polarisation phenotype of TAMs is associated with survival outcomes independent of potential confounding factors. More specifically, higher stromal densities of M2-like macrophages and lower M1:M2 macrophage polarisation ratios in the stromal and epithelial compartments in the invasive margins of the pulmonary metastases are associated with worse survival.

In the early phase of the inflammatory response, the extravasated monocytes and tissue-resident macrophages are frequently transformed into pro-inflammatory, phagocytic M1-oriented macrophages which have generally been associated with better cancer prognosis [2]. In the later phases of the inflammatory response, macrophages shift to a more anti-inflammatory M2-like phenotype which orchestrates wound-healing mechanisms such as angiogenesis, matrix deposition, immunosuppression, and tissue remodelling [2]. If prolonged, these mechanisms may be utilised by tumour cells for growth promotion and metastasis [33]. Despite the M1/M2 polarisation concept in macrophage phenotyping being an oversimplification that overlooks other recognized macrophage subpopulations [34, 35], it has provided a useful framework for macrophage phenotyping. Indeed, several meta-analyses on different cancer types support the prognostic value of M1/M2-like macrophages [36–38]. In CRC, two recent meta-analyses concluded overall TAMs being associated with improved survival [7, 8], the association being more prominent in microsatellite stable patients [7]. Higher densities of M2-like macrophages have been associated with poor survival [9, 12], M1-like macrophages with favourable clinical outcomes [12], and, accordingly, higher M1:M2 ratios with longer survival [10]. However, contradictory results have also been reported [12, 13]. TAMs in primary CRC tumours have been found to be more M2-polarised in higher disease stages [9]. In the metastatic setting, the prognostic effect of TAMs is less well-understood. A recent article demonstrated the morphology of CD163^+^ TAMs being a prognostic factor in CRC liver metastasis, whereas the density of CD163^+^ TAMs was not associated with prognosis [21]. Also, Takahashi et al. showed CD206^+^ TAMs associating with shorter disease-free survival [20], whereas CD68^+^ TAMs have been reported to be associated with longer disease-free survival [39]. We found no previous studies on the prognostic role of TAMs in CRC pulmonary metastases.

Our study shows a significant decrease in M1:M2 density ratios and a more M2-polarised phenotype of TAMs in the pulmonary metastases in comparison to the primary tumours. Higher densities of M2-like macrophages in the invasive margin of pulmonary metastases were associated with worse prognosis, while the overall macrophage population did not harbour prognostic significance. The negative prognostic effect of high epithelial M1-like macrophage densities in the centre of the pulmonary metastases in our data might be coincidental due to the small epithelial macrophage densities and the uneven stratification into high/low groups (75% of the cases in high category). Using median cutoffs, the epithelial M1-like macrophage densities were not prognostic. Furthermore, higher M1:M2 density ratios in the invasive margin of pulmonary metastases were associated with favourable prognosis. High intratumoural heterogeneity of stromal M2-like macrophage densities in pulmonary metastases was also associated with worse survival, and the heterogeneity was higher in the metastases compared to the primary tumours. The integrative macrophage polarisation score did not provide additional prognostic value, which underlines the significance of macrophage densities within specific tumour regions that can be evaluated by spatially informed methods such as multiplex immunohistochemistry. Pulmonary metastases are known to be more immunogenic in comparison to brain, liver, or bone metastases [40], which might account for the difference in the prognostic value of TAMs in our study compared to earlier studies evaluating CRC liver metastases. On the other hand, the scarce literature on TAMs in CRC metastases in general and the single-macrophage marker basis of most of the previous studies might also account for the difference. Nevertheless, our results support the prognostic significance of M2-like macrophages and highlight the rationale of multi-marker analysis in identifying cell populations that show higher prognostic relevance than those defined by a single macrophage marker.

Reference literature has demonstrated a decrease in overall macrophage densities in the primary tumours alongside the increase of stage [13, 41] and liver metastasis [10]. In our data, there was a significant increase in overall macrophage densities in the invasive margin of the pulmonary metastases compared to the primary tumours, whereas the densities in the tumour centres were similar. We also evaluated TAM polarisation, and the M1:M2 density ratios shifted towards a more M2-polarised state in the metastases. In spatial analysis, the macrophages were closer to cancer cells in the first resected pulmonary metastases compared to the primary tumours; the M1 macrophages were closer to cancer cells than M2 macrophages, as was with the primary tumours. Taken together, it seems that during cancer progression and metastasis, macrophages transform to a more pro-tumoural M2-like phenotype while moving closer to the cancer cells.

Beyond contributing to cancer growth and invasion, macrophages are also proposed to account for the efficacy of adjuvant therapy. Neoadjuvant therapy has been suggested to shift TAM polarisation towards an anti-tumoural M1-like oriented state in primary colorectal and pancreatic tumours [3, 4]. Also, TAMs have been proposed to contribute to the immune checkpoint therapy efficacy, as PD-L1 expression is higher in TAMs than in tumour cells [24, 42]. On the other hand, M2-polarised macrophages may mediate chemotherapy resistance [5]. In our study, neoadjuvant therapy was associated with a shift in TAM polarisation to a more M1-like oriented state in primary tumours, although there might be a major selection bias of primary tumours in our study. Instead, neoadjuvant therapy was not associated with TAM polarisation in pulmonary metastases. Also, in the sensitivity analysis including only patients without neoadjuvant treatment, TAM densities did not provide additional prognostic value, which might suggest shifted immune milieu in the metastases compared to the primary tumours, but also the reduced sample size and statistical power in this analysis might have an impact on the result. The association between neoadjuvant therapy resistance and TAMs is seen also in the spatial analysis of TAMs. A recent multiplexed image analysis of the immune contexture in hepatocellular cancers in patients treated with neoadjuvant vascular endothelial growth factor and PD-1 inhibition revealed that non-responders and responders had significant differences in the spatial relations of TAMs, lymphocytes, and tumour cells [43]. The TAMs and lymphocytes had strong communication with the tumour cells in the responders, while in the non-responders, the cytotoxic CD8^+^ T-cells were in close proximity with immunosuppressive arginase 1 and CCR6 expressing CD163^−^ TAMs. Furthermore, a study on pancreatic cancer reported that a close proximity of immunosuppressive IL10^+^ myelomonocytes to cytotoxic granzyme-B^+^ CD8^+^ T-cells instead of PD-1^+^ CD4 + T-cells was associated with shorter survival in non-neoadjuvant treated patients [44]. In a validation cohort in the study including only neoadjuvant-treated patients, the IL10^+^ myelomonocytes were shifted closer to the PD-1^+^ CD4^+^ T-cells demonstrating possible spatial changes of inhibited immunosuppression after neoadjuvant chemoradiotherapy. In our study, focusing the analysis on the polarisation state of TAMs using multi-marker phenotyping, anti-tumoural M1-like macrophages were located closer to tumour cells compared to pro-tumoural M2-like macrophages. Altogether, these results could be interpreted that the pro-tumoural activity of immunosuppressive macrophages might occur distant from the tumour cells and in closer contact with other cells in the TME. Our results also showed that higher intratumoural M2-like macrophage heterogeneity is associated with worse survival. Reference literature indicates that intratumoural spatial heterogeneity (alongside other measures of heterogeneity) is a hallmark in adjuvant therapy resistance [45]. In demonstrating the prognostic significance of the spatial nuances of the immune contexture in different cancer types, the multiplexed analysis has shown potential in search for new targets for future cancer therapy. Further studies on the spatial analysis of the immune contexture in cancer metastases would deepen the understanding of mechanisms of metastatic dissemination.

TAMs themselves have also emerged as a promising target for adjuvant therapy; however, the promising results in preclinical studies of several therapeutic agents in TAM-targeted treatment have not been equally satisfactory in clinical studies and the subgroups of cancer patient benefitting from TAM-targeted therapy are yet to be found [16]. In nanoimmunotherapy, recent advances are focusing on the termination of macrophage recruitment to the TME, repolarisation of M2 macrophages to M1 macrophages, and interference of TAM survival [46]. For instance, RRx-001 is a small molecule immunotherapeutic agent planned to overrule chemotherapy resistance by causing TAM repolarisation for M2 macrophages to M1 macrophages and is going through phase III clinical trials on small cell lung cancer [47]. It remains to be seen, whether metastatic CRC might benefit from TAM targeting adjuvant therapy, considering the more M2-like oriented state of TAMs in CRC pulmonary metastases compared to the primary tumours in our study.

There are some limitations in our study. First, the surgical patient selection for pulmonary metastasectomy represents a considerable selection bias, and our results cannot be generalised to all pulmonary metastases without precautions. Second, the use of TMAs is a limitation, as the cell densities in small tissue samples do not necessarily represent cell densities in the whole slide. However, TMA-based analysis has offered reproducible results in former studies [48]. Third, adjuvant therapy data were not available, and different adjuvant treatment regimens might have affected the survival of our study patients. Fourth, the lack of data on RAS mutation status can be considered a limitation. However, due to the long study period, at the beginning of which the RAS mutation status was not extensively used in clinical decision making, the post hoc determination of RAS status was not performed. The lack of macrophage density analysis in other resected metastases in cases where more than one metastasis was resected also limits the added value of this study, as they might also have contained relevant data on TAM biology. Last, the study period encompasses 20 years during which the diagnostics and treatment might have evolved, possibly affecting our results. Concerning the absence of MMR deficient tumours in our cohort, the proportion of MMR deficiency in CRC cohorts of all stages is around 15–20% [49], whereas in stage IV CRC cohorts it is much lower, 4–5% [50]. In CRC pulmonary metastasectomy cohorts, a proportion of 0% has been previously reported [51]. As all of our tumours were MMR proficient (and thus likely microsatellite stable), our results cannot be generalised to CRC patients with microsatellite unstable tumours. The strengths of the study include a reasonably sized, dual-institutional cohort of CRC pulmonary metastases. The histopathological features and prognostic factors of CRC pulmonary metastases have not been extensively studied, and to the best of our knowledge, there are no previous studies on the significance of TAMs in CRC pulmonary metastases. The use of multi-marker phenotyping of TAMs is a strength, as single macrophage markers are known to overlap between macrophage phenotypes and other cell types, accounting for the reproducibility challenge in TAM literature [14]. The use of machine learning techniques in digital image analysis also represents a considerable strength as it provides more precise estimates of macrophage densities and the localisation of individual macrophages in comparison to semi-quantitative visual estimates [52].

## Conclusion

In conclusion, macrophages in CRC pulmonary metastases show altered (more M2-like) polarisation phenotype as compared to the primary tumours. Higher densities of stromal M2-like macrophages and lower M1:M2 macrophage polarisation ratios in the invasive margin of the pulmonary metastases are associated with worse overall survival. The results highlight the value of multiplexed analysis of macrophage polarisation phenotypes in tumour metastasis and might have clinical implications in future cancer therapy.

### Supplementary Information

Below is the link to the electronic supplementary material.

Supplementary file 1 (DOCX 765 kb)

## Data Availability

Data generated and/or analysed during this study are not publicly available. The sharing of data will require approval from relevant ethics committees and/or biobanks. Further information including the procedures to obtain and access data from Finnish Biobanks are described at https://finbb.fi/en/fingenious-service
