# Supplementary material for: Multiplexed analysis of macrophage polarisation in pulmonary metastases of microsatellite stable colorectal cancer
Source: Cancer Immunol Immunother. 2024 Feb 22;73(3):59. doi: 10.1007/s00262-024-03646-0 (PMC10884151; doi:10.1007/s00262-024-03646-0)
Supplement: Supplementary file 1 — (DOCX 765 kb) [file 262_2024_3646_MOESM1_ESM.docx]

**Supplementary Material**

**Table S1.** Cut-offs for M1- and M2-like macrophage densities and M1:M2 polarisation ratios selected based on ROC analysis.

|  | Epithelial TC | Epithelial IM | Stromal TC | Stromal IM | Overall |
| --- | --- | --- | --- | --- | --- |
| M1-like macrophages | 16.636 | 52.789 | 132.122 | 198.29 | 118.22 |
| M2-like macrophages | 28.57 | 33.56 | 181.48 | 278.64 | 93.70 |
| M1:M2 ratio | 2.412 | 2.674 | 0.616 | 0.858 | 0.619 |

IM=invasive margin; TC=tumour centre

**Table S2.** Unadjusted hazard ratios for various clinical parameters in 5-year overall survival (after pulmonary metastasectomy) Cox proportional hazard regression models.

| Variable | HR (95% CI) | *p-*value |
| --- | --- | --- |
| Gender |  | *p*=0.384 |
| Female | 1.00 (reference) |  |
| Male | 1.32 (0.71-2.49) |  |
| RCSCS |  | *p*=0.956 |
| 1 | 1.00 (reference) |  |
| 2 | 0.89 (0.40-1.97) |  |
| ≥3 | 1.00 (0.41-2.46) |  |
| Age (continuous) | 1.03 (1.0-1.06) | *p=*0.071 |
| Neoadjuvant therapy |  | *p*=0.908 |
| No | 1.00 (reference) |  |
| Yes | 1.04 (0.55-1.96) |  |
| Former liver metastases |  | *p*=0.172 |
| No | 1.00 (reference) |  |
| Yes | 0.64 (0.34-1.21) |  |
| Number of pulmonary metastases at diagnosis |  | *p=*0.471 |
| 1 | 1.00 (reference) |  |
| ≥1 | 1.27 (0.67-2.40) |  |
| Synchronicity |  | *p=*0.366 |
| Synchronous | 1.00 (reference) |  |
| Metachronous | 0.68 (0.3-1.56) |  |

RCSCS= Royal College of Surgeons Comorbidity Score

**Table S3**. Macrophage densities and polarisation according to the order of the pulmonary metastasectomies.

|  | Order of pulmonary metastasectomy | |  |
| --- | --- | --- | --- |
|  | **First** | **Later** |  |
|  | **MD (IQR)** | **MD (IQR)** | ***p*** |
| Tumour centre | | | |
| Stromal macrophages | | | |
| M1-like | 153.7 (102.6-309.8) | 132.8 (73.9-272.9) | 0.297 |
| M2-like | 229.7 (113.0-379.9) | 208.2 (113.5-308.9) | 0.707 |
| M1:M2 ratio | 0.6 (0.3-2.3) | 0.7 (0.3-1.4) | 0.964 |
| Overall (CD68) | 977.9 (714.4-1359.2) | 1003.6 (570.0-1254.5) | 0.400 |
| Epithelial macrophages | | | |
| M1-like | 117.2 (44.9-218.0) | 102.3 (39.5-200.8) | 0.317 |
| M2-like | 75.1 (17.3-200.4) | 62.0 (13.6-176.6) | 0.272 |
| M1:M2 ratio | 1.5 (0.5-5.5) | 1.8 (0.6-5.6) | 0.651 |
| Overall (CD68) | 137.3 (65.0-352.8) | 74.8 (34.8-148.9) | **0.022*** |
| Overall (stromal + epithelial) macrophages | | | |
| M1-like | 106.1 (48.6-179.7) | 55.6 (31.6-117.2) | 0.059 |
| M2-like | 94.2 (53.4-164.8) | 81.8 (36.4-167.5) | 0.311 |
| M1:M2 ratio | 0.78 (0.35-2.38) | 0.8 (0.3-1.7) | 0.610 |
| Overall (CD68) | 504.9 (307.1-813.4) | 324.7 (196.3-604.5) | **0.050*** |
| Invasive margin | | | |
| Stromal macrophages | | | |
| M1-like | 191.7 (125.0-248.2) | 196.5 (154.1-321.5) | 0.611 |
| M2-like | 285.2 (170.3-425.0) | 255.1 (95.5-483.8) | 0.403 |
| M1:M2 ratio | 0.6 (0.3-1.5) | 0.7 (0.3-1.8) | 0.420 |
| Overall (CD68) | 1181.1 (935.3-1803.5) | 1130.6 (869.3-1474.5) | 0.386 |
| Epithelial macrophages | | | |
| M1-like | 81.9 (31.0-167.3) | 56.9 (34.9-171.1) | 0.788 |
| M2-like | 39.4 (15.3-125.2) | 29.9 (6.3-77.7) | 0.455 |
| M1:M2 ratio | 1.7 (0.6-4.6) | 2.4 (0.9-7.0) | 0.339 |
| Overall (CD68) | 370.3 (105.4-535.2) | 309.8 (115.4-498.8) | 0.698 |
| Overall (stromal + epithelial) macrophages | | | |
| M1 | 145.2 (72.5-196.6) | 117.8 (76.5-190.3) | 0.543 |
| M2 | 160.1 (77.1-291.6) | 100.0 (46.9-268.7) | 0.169 |
| M1:M2 ratio | 0.8 (0.3-1.9) | 0.8 (0.4-2.7) | 0.408 |
| Overall (CD68) | 757.6 (528.7-1169.7) | 645.5 (382.7-791.2) | 0.127 |

*statistically significant at the level *p*<0.05

**Table S4**. Spearman correlation coefficients of M1- and M2-like macrophages in the first resected pulmonary metastases of colorectal carcinoma

|  | | | M1-like macrophages | | | | M2-like macrophages | | | |
| --- | --- | --- | --- | --- | --- | --- | --- | --- | --- | --- |
|  |  |  | Epith TC | Epith IM | Strom TC | Strom IM | Epith TC | Epith IM | Strom TC | Strom IM |
| M1-like macrophages | Epithelial TC | r_s_ | 1.000 | **0.348**** | **0.666**** | **0.288*** | **0.340**** | 0.099 | -0.038 | -0.114 |
|  |  | *p* |  | <0.001 | <0.001 | 0.035 | <0.001 | 0.478 | 0.775 | 0.411 |
|  |  | N | 636 | 54 | 60 | 54 | 636 | 54 | 60 | 54 |
|  | Epithelial IM | r_s_ | **0.348**** | 1.000 | **0.296*** | **0.532**** | 0.221 | **0.478**** | 0.123 | 0.010 |
|  |  | *p* | <0.001 |  | <0.001 | <0.001 | 0.108 | <0.001 | 0.376 | 0.464 |
|  |  | N | 54 | 56 | 54 | 56 | 54 | 56 | 54 | 56 |
|  | Stromal TC | r_s_ | **0.666**** | **0.296*** | 1.000 | **0.297*** | -0.123 | -0.010 | **-0.291*** | -0.169 |
|  |  | *p* | <0.001 | 0.030 |  | 0.029 | 0.348 | 0.942 | 0.024 | 0.223 |
|  |  | N | 60 | 54 | 60 | 54 | 60 | 54 | 60 | 54 |
|  | Stromal IM | r_s_ | **0.288*** | **0.532**** | **0.297*** | 1.000 | -0.039 | -0.002 | **-0.291*** | **-0.324*** |
|  |  | *p* | 0.035 | <0.001 | 0.029 |  | 0.777 | 0.987 | 0.033 | 0.015 |
|  |  | N | 54 | 56 | 54 | 56 | 54 | 56 | 54 | 56 |
| M2-like macrophages | Epithelial TC | r_s_ | **0.340**** | 0.221 | -0.123 | -0.039 | 1.000 | **0.480**** | **0.511**** | **0.399**** |
|  |  | *p* | <0.001 | 0.108 | 0.348 | 0.777 |  | <0.001 | <0.001 | 0.003 |
|  |  | N | 636 | 54 | 60 | 54 | 636 | 54 | 60 | 54 |
|  | Epithelial IM | r_s_ | 0.099 | **0.478**** | -0.010 | -0.002 | **0.480**** | 1.000 | **0.365**** | **0.642**** |
|  |  | *p* | 0.478 | <0.001 | 0.942 | 0.987 | <0.001 |  | 0.007 | <0.001 |
|  |  | N | 54 | 56 | 54 | 56 | 54 | 56 | 54 | 56 |
|  | Stromal TC | r_s_ | -0.038 | 0.123 | **-0.291*** | **-0.291*** | **0.511**** | **0.365**** | 1.000 | **0.554**** |
|  |  | *p* | 0.775 | 0.376 | 0.024 | 0.033 | <0.001 | 0.007 |  | <0.001 |
|  |  | N | 60 | 54 | 60 | 54 | 60 | 54 | 60 | 54 |
|  | Stromal IM | r_s_ | -0.114 | 0.010 | -0.169 | **-0.324*** | **0.399**** | **0.642**** | **0.554**** | 1.000 |
|  |  | *p* | 0.411 | 0.464 | 0.223 | 0.015 | 0.003 | <0.001 | <0.001 |  |
|  |  | N | 54 | 56 | 54 | 56 | 54 | 56 | 54 | 56 |

Epith=epithelial; IM=invasive margin; r_s_=Spearman correlation coefficient; Strom=stromal; TC=tumour centre

** statistically significant at the level *p*<0.01

* statistically significant at the level *p*<0.05

**Table S5**. Spearman correlation analysis of M1- and M2-like macrophage densities in the first resected pulmonary metastases and corresponding primary CRC tumours.

|  | | | | Primary tumours | | | | | | | |
| --- | --- | --- | --- | --- | --- | --- | --- | --- | --- | --- | --- |
|  |  |  |  | **M1-like macrophages** | | | | **M2-like macrophages** | | | |
|  |  |  |  | Epith TC | Epith IM | Strom TC | Strom IM | Epith TC | Epith IM | Strom TC | Strom IM |
| Pulmonary metastases | **M1-like macrophages** | Epithelial TC | r_s_ | **0.295**** | -0.088 | -0.063 | -0.033 | -0.089 | -0.106 | -0.160 | 0.009 |
|  |  |  | *p* | <0.001 | 0.584 | 0.673 | 0.837 | 0.553 | 0.509 | 0.283 | 0.957 |
|  |  |  | N | 255 | 41 | 47 | 41 | 47 | 41 | 47 | 41 |
|  |  | Epithelial IM | r_s_ | 0.213 | 0.164 | 0.115 | 0.256 | -0.045 | -0.012 | 0.009 | -0.101 |
|  |  |  | *p* | 0.171 | 0.325 | 0.463 | 0.121 | 0.775 | 0.945 | 0.952 | 0.546 |
|  |  |  | N | 43 | 38 | 43 | 38 | 43 | 38 | 43 | 38 |
|  |  | Stromal TC | r_s_ | -0.098 | 0.068 | -0.025 | 0.105 | -0.162 | 0.081 | -0.130 | -0.008 |
|  |  |  | *p* | 0.512 | 0.673 | 0.865 | 0.515 | 0.276 | 0.614 | 0.384 | 0.959 |
|  |  |  | N | 47 | 41 | 47 | 41 | 47 | 41 | 47 | 41 |
|  |  | Stromal IM | r_s_ | 0.129 | 0.088 | 0.154 | 0.070 | -0.254 | -0.128 | -0.226 | -0.154 |
|  |  |  | *p* | 0.408 | 0.601 | 0.325 | 0.675 | 0.100 | 0.444 | 0.145 | 0.357 |
|  |  |  | N | 43 | 38 | 43 | 38 | 43 | 38 | 43 | 38 |
|  | **M2-like macrophages** | Epithelial TC | r_s_ | **0.419**** | -0.232 | -0.110 | -0.134 | 0.020 | -0.237 | -0.038 | -0.020 |
|  |  |  | *p* | <0.001 | 0.145 | 0.463 | 0.404 | 0.894 | 0.135 | 0.801 | 0.900 |
|  |  |  | N | 255 | 41 | 47 | 41 | 47 | 41 | 47 | 41 |
|  |  | Epithelial IM | r_s_ | 0.046 | 0.114 | 0.083 | 0.224 | 0.054 | -0.114 | 0.042 | -0.137 |
|  |  |  | *p* | 0.768 | 0.497 | 0.598 | 0.177 | 0.732 | 0.495 | 0.787 | 0.412 |
|  |  |  | N | 43 | 38 | 43 | 38 | 43 | 38 | 43 | 38 |
|  |  | Stromal TC | r_s_ | 0.034 | -0.143 | -0.162 | 0.056 | 0.031 | -0.128 | 0.251 | 0.129 |
|  |  |  | *p* | 0.821 | 0.373 | 0.278 | 0.730 | 0.838 | 0.426 | 0.089 | 0.422 |
|  |  |  | N | 47 | 41 | 47 | 41 | 47 | 41 | 47 | 41 |
|  |  | Stromal IM | r_s_ | -0.036 | 0.063 | -0.019 | 0.232 | -0.037 | -0.251 | -0.052 | 0.025 |
|  |  |  | *p* | 0.821 | 0.706 | 0.904 | 0.161 | 0.811 | 0.128 | 0.739 | 0.879 |
|  |  |  | N | 43 | 38 | 43 | 38 | 43 | 38 | 43 | 38 |

Epith=epithelial; IM=invasive margin; r_s_=Spearman correlation coefficient; Strom=stromal; TC=tumour centre

*statistically significant at the level *p*<0.05

**Table S6.** Hazard ratios with 95% confidence intervals for 5-year all-cause mortality according to M1- and M2-like macrophage densities and M1:M2 ratios in the first pulmonary metastases in patients not receiving neoadjuvant therapy.

|  | M1-like macrophage density | | M2-like macrophage density | | M1:M2 ratio | |
| --- | --- | --- | --- | --- | --- | --- |
|  | **Low,** HR (95%CI) | **High,** HR (95%CI) | **Low,** HR (95%CI) | **High,** HR (95%CI) | **Low,** HR (95%CI) | **High,** HR (95%CI) |
| Tumour centre | | | | | | |
| Stomal | | | | | | |
| Crude | 1.00 (reference) | 0.99 (0.38-2.58; *p*=0.980) | 1.00 (reference) | 0.60 (0.24-1.50; *p*=0.269) | 1.00 (reference) | 1.22 (0.48-3.07; *p*=0.680) |
| Adjusted* | 1.00 (reference) | 0.95 (0.33-2.75; *p*=0.931) | 1.00 (reference) | 0.64 (0.21-1.92; *p*=0.422) | 1.00 (reference) | 1.02 (0.39-2.67; *p*=0.965) |
| Epithelial | | | | | | |
| Crude | 1.00 (reference) | 1.96 (0.57-6.74; *p*=0.285) | 1.00 (reference) | 1.46 (0.60-3.53; *p*=0.402) | 1.00 (reference) | 1.22 (0.50-2.98; *p*=0.699) |
| Adjusted* | 1.00 (reference) | 1.66 (0.47-5.91; *p*=0.432) | 1.00 (reference) | 1.70 (0.60-4.84; *p=*0.322) | 1.00 (reference) | 1.05 (0.40-2.80; *p*=0.916) |
| Invasive margin | | | | | | |
| Stromal | | | | | | |
| Crude | 1.00 (reference) | 0.44 (0.16-1.20; *p=*0.110) | 1.00 (reference) | 1.78 (0.67-4.73; *p*=0.244) | 1.00 (reference) | 0.40 (0.14-1.14; *p*=0.085) |
| Adjusted* | 1.00 (reference) | 0.46 (0.16-1.34; *p*=0.154) | 1.00 (reference) | 2.08 (0.76-5.72; *p*=0.154) | 1.00 (reference) | 0.22 (0.055-0.89; ***p*=0.034**) |
| Epithelial | | | | | | |
| Crude | 1.00 (reference) | 2.30 (0.52-10.05; *p*=0.270) | 1.00 (reference) | 1.84 (0.60-5.66; *p*=0.288) | 1.00 (reference) | 0.45 (0.16-1.28; *p*=0.135) |
| Adjusted* | 1.00 (reference) | 4.12 (0.76-22.36; *p*=0.101) | 1.00 (reference) | 1.40 (0.42-4.70; *p*=0.588) | 1.00 (reference) | 0.25 (0.06-1.17; *p*=0.078) |

*Cox proportional hazards regression models adjusted for gender (female/male), age (continuous), RCSCS (1/2/≥3), neoadjuvant therapy (no/yes), synchronicity of pulmonary metastases (synchronous/metachronous), number of pulmonary metastases at diagnosis (1/≥1) former liver metastases (no/yes).

**Table S7** Baseline characteristics of colorectal cancer cases according to epithelial M1- and M2-like macrophage densities in the invasive margin of the first resected pulmonary metastases.

|  | M1-like macrophages | |  | M2-like macrophages | |  |
| --- | --- | --- | --- | --- | --- | --- |
|  | **Low** | **High** | ***p*** | **Low** | **High** | ***p*** |
|  | n (%) | n (%) |  | n (%) | n (%) |  |
| n | 19 | 37 |  | 24 | 32 |  |
| Sex |  |  | 0.397 |  |  | 0.280 |
| Female | 11 (57.9%) | 17 (45.9%) |  | 14 (58.3%) | 14 (43.8%) |  |
| Male | 8 (42.1%) | 20 (54.1%) |  | 10 (41.7%) | 18 (56.3%) |  |
| Age (M; SD) | 68.72 (11.59) | 68.72 (11.59) | 0.272 | 68.72 (11.59) | 68.72 (11.59) | 0.202 |
| RCSCS |  |  | 0.206 |  |  | 0.178 |
| 1 | 10 (52.6%) | 24 (64.9%) |  | 14 (58.3%) | 20 (62.5%) |  |
| 2 | 7 (36.8%) | 6 (16.2%) |  | 8 (33.3%) | 5 (15.6%) |  |
| ≥3 | 2 (10.5%) | 7 (18.9%) |  | 2 (8.3%) | 7 (21.9%) |  |
| Neoadjuvant chemotherapy |  |  | 0.143 |  |  | 0.155 |
| No | 9 (47.4%) | 25 (67.6%) |  | 12 (50.0%) | 22 (68.8%) |  |
| Yes | 10 (52.6%) | 12 (32.4%) |  | 12 (50.0%) | 10 (31.3%) |  |
| Disease stage |  |  | 0.313 |  |  | 0.241 |
| 1-2 | 5 (26.3%) | 16 (43.2%) |  | 6 (25.0%) | 15 (46.9%) |  |
| 3 | 10 (52.6%) | 12 (32.4%) |  | 11 (45.8%) | 11 (34.4%) |  |
| 4 | 4 (21.1%) | 9 (24.3%) |  | 7 (29.2%) | 6 (18.8%) |  |
| Primary tumour location |  |  | 0.778 |  |  | >0.999 |
| Colon | 10 (52.6%) | 18 (48.6%) |  | 12 (50.0%) | 16 (50.0%) |  |
| Rectum | 9 (47.4%) | 19 (51.4%) |  | 12 (50.0%) | 16 (50.0%) |  |
| Disease-free interval (d; MD; IQR) | 309 (0-925) | 427 (67-857) | 0.811 | 311.5 (0-869) | 627.5 (110-932) | 0.266 |
| Size of largest pulmonary metastasis (cm; MD; IQR) | 1.5 (1.2-2.7) | 2.5 (1.4-3.6) | 0.080 | 2 (1.05-3.5) | 2.4 (1.5-3.5) | 0.203 |
| Former liver metastases |  |  | 0.769 |  |  | 0.485 |
| No | 10 (52.6%) | 21 (56.8%) |  | 12 (50.0%) | 19 (59.4%) |  |
| Yes | 9 (47.4%) | 16 (43.2%) |  | 12 (50.0%) | 13 (40.6%) |  |
| Synchronicity |  |  | 0.523 |  |  | 0.222 |
| Synchronous | 5 (26.3%) | 7 (18.9%) |  | 7 (29.2%) | 5 (15.6%) |  |
| Metachronous | 14 (73.7%) | 30 (81.1%) |  | 17 (70.8%) | 27 (84.4%) |  |
| No. of pulmonary metastases |  |  | 0.399 |  |  | 0.719 |
| 1 | 10 (52.6%) | 24 (64.9%) |  | 14 (58.3%) | 20 (62.5%) |  |
| 2 | 6 (31.6%) | 11 /29.7%) |  | 7 (29.2%) | 10 (31.3%) |  |
| ≥3 | 3 (15.8%) | 2 (5.4%) |  | 3 (12.5%) | 2 (6.3%) |  |
| Laterality of metastases |  |  | 0.961 |  |  | 0.452 |
| Unilateral | 15 (78.9%) | 29 (78.4%) |  | 20 (83.3%) | 24 (75.0%) |  |
| Bilateral | 4 (21.1%) | 8 (21.6%) |  | 4 (16.7%) | 8 (25.0%) |  |
| *BRAF* |  |  | 0.322 |  |  | 0.760 |
| Wild-type | 17 (100.0%) | 34 (94.4%) |  | 20 (95.2%) | 31 (96.9%) |  |
| Mutant | 0 (0.0%) | 2 (5.6%) |  | 1 (4.8%) | 1 (3.1%) |  |

RCSCS= Royal College of Surgeons Comorbidity Score. The chi-square test, Student’s T-test, and Mann-Whitney U-test were applied.

**Table S8.** Baseline characteristics according to M1:M2 density ratio in the invasive margin of CRC pulmonary metastases.

|  | M1:M2 ratio, epithelial | |  | M1:M2 ratio, stromal | |  |
| --- | --- | --- | --- | --- | --- | --- |
|  | Low | High |  | Low | High |  |
|  | n (%) | n (%) | *p* | n (%) | n (%) | *p* |
| n | 31 | 24 |  | 33 | 23 |  |
| Sex |  |  | 0.508 |  |  | 0.786 |
| Female | 17 (54.8%) | 11 (45.8%) |  | 16 (48.5%) | 12 (52.2%) |  |
| Male | 14 (45.2%) | 13 (54.2%) |  | 17 (51.5%) | 11 (47.8%) |  |
| Age (M; SD) | 67.32 (11.25) | 67.5 (10.35) | 0.952 | 68.24 (11.24) | 66.22 (9.91) | 0.490 |
| RCSCS |  |  | 0.482 |  |  | 0.500 |
| 1 | 21 (67.7%) | 13 (54.2%) |  | 22 (66.7%) | 12 (52.2%) |  |
| 2 | 5 (16.1%) | 7 (29.2%) |  | 6 (18.2%) | 7 (30.4%) |  |
| ≥3 | 5 (16.1%) | 4 (16.7%) |  | 5 (15.2%) | 4 (17.4%) |  |
| Neoadjuvant chemotherapy |  |  | 0.437 |  |  | 0.275 |
| No | 20 (64.5%) | 13 (54.2%) |  | 22 (66.7%) | 12 (52.2%) |  |
| Yes | 11 (35.5%) | 11 (45.8%) |  | 11 (33.3%) | 11 (47.8%) |  |
| Disease stage |  |  | 0.208 |  |  | 0.431 |
| 1-2 | 15 (48.4%) | 6 (25.0%) |  | 12 (36.4%) | 9 (39.1%) |  |
| 3 | 10 (32.3%) | 11 (45.8%) |  | 15 (45.5%) | 7 (30.4%) |  |
| 4 | 6 (19.4%) | 7 (29.2%) |  | 6 (18.2%) | 7 (30.4%) |  |
| Primary tumour location |  |  | 0.906 |  |  | 0.415 |
| Colon | 15 (48.4%) | 12 (50.0%) |  | 18 (54.5%) | 10 (43.5%) |  |
| Rectum | 16 (51.6%) | 12 (50.0%) |  | 15 (45.5%) | 13 (56.5%) |  |
| Disease-free interval (d; MD; IQR) | 482 (67-857) | 325 (0-990.5) | 0.777 | 621 (77-813) | 314 (0-1004) | 0.619 |
| Size of largest pulmonary metastasis (cm; MD; IQR) | 2.5 (1.5-3.5) | 2 (1-3.5) | 0.320 | 2.5 (1.5-3.5) | 2 (1-3.5) | 0.314 |
| Former liver metastases |  |  | 0.419 |  |  | 0.884 |
| No | 16 (51.6%) | 15 (62.5%) |  | 18 (54.5%) | 13 (56.5%) |  |
| Yes | 15 (48.4%) | 9 (37.5%) |  | 15 (45.5%) | 10 (43.5%) |  |
| Synchronicity |  |  | 0.069 |  |  | 0.478 |
| Synchronous | 4 (12.9%) | 8 (33.3%) |  | 6 (18.2%) | 6 (26.1%) |  |
| Metachronous | 27 (87.1%) | 16 (66.7%) |  | 27 (81.8%) | 17 (73.9%) |  |
| No. of pulmonary metastases |  |  | 0.127 |  |  | 0.999 |
| 1 | 15 (48.4%) | 18 (75.0%) |  | 20 (60.6%) | 14 (60.9%) |  |
| 2 | 12 (38.7%) | 5 (20.8%) |  | 10 (30.3%) | 7 (30.4%) |  |
| ≥3 | 4 (12.9%) | 1 (4.2%) |  | 3 (9.1%) | 2 (8.7%) |  |
| Laterality of metastases |  |  | 0.416 |  |  | 0.962 |
| Unilateral | 23 (74.2%) | 20 (83.3%) |  | 26 (78.8%) | 18 (78.3%) |  |
| Bilateral | 8 (25.8%) | 4 (16.7%) |  | 7 (21.2%) | 5 (21.7%) |  |
| *BRAF* |  |  | 0.092 |  |  | 0.075 |
| Wild-type | 30 (100.0%) | 20 (90.9%) |  | 32 (100.0%) | 19 (90.5%) |  |
| Mutant | 0 (0.0%) | 2 (9.1%) |  | 0 (0.0%) | 2 (9.5%) |  |

RCSCS=Royal College of Surgeons Comorbidity Score. The chi-square test, Student’s T-test, and Mann-Whitney U-test were applied.

**Table S9**. Baseline characteristics according to epithelial M1- and M2-like macrophage densities in the invasive margin of primary tumours.

|  | M1-like macrophages | |  | M2-like macrophages | |  |
| --- | --- | --- | --- | --- | --- | --- |
|  | **Low** | **High** |  | **Low** | **High** |  |
|  | n (%) | n (%) | p | n (%) | n (%) | p |
| n | 29 | 17 |  | 28 | 18 |  |
| Sex |  |  | 0.708 |  |  | 0.474 |
| Female | 17 (59%) | 9 (53%) |  | 17 (61%) | 9 (50%) |  |
| Male | 12 (41%) | 8 (47%) |  | 11 (39%) | 9 (50%) |  |
| Age (M; SD) | 69.69 (9.14) | 64.88 (12.55) | 0.141 | 67.93 (11.16) | 67.89 (10.14) | 0.990 |
| RCSCS |  |  | 0.247 |  |  | 0.125 |
| 1 | 17 (59%) | 14 (82%) |  | 16 (57%) | 15 (83%) |  |
| 2 | 7 (24%) | 2 (12%) |  | 8 (29%) | 1 (6%) |  |
| ≥3 | 5 (17%) | 1 (6%) |  | 4 (14%) | 2 (11%) |  |
| Neoadjuvant chemotherapy |  |  | **0.002**** |  |  | 0.464 |
| No | 26 (93%) | 9 (53%) |  | 22 (82%) | 13 (72%) |  |
| Yes | 2 (7%) | 8 (47%) |  | 5 (19%) | 5 (28%) |  |
| Disease stage |  |  | **0.037*** |  |  | 0.506 |
| 1–2 | 10 (35%) | 8 (47%) |  | 10 (36%) | 8 (44%) |  |
| 3 | 10 (35%) | 9 (53%) |  | 11 (39%) | 8 (44%) |  |
| 4 | 9 (31%) | 0 (0%) |  | 7 (25%) | 2 (11%) |  |
| Primary tumour location |  |  | **0.002**** |  |  | 0.337 |
| Colon | 22 (76%) | 5 (30%) |  | 18 (64%) | 9 (50%) |  |
| Rectum | 7 (24%) | 12 (71%) |  | 10 (36%) | 9 (50%) |  |
| Primary tumour grade |  |  | 0.258 |  |  | 0.922 |
| 1 | 8 (31%) | 6 (35%) |  | 8 (31%) | 6 (35%) |  |
| 2 | 16 (62%) | 7 (41%) |  | 14 (54%) | 9 (53%) |  |
| 3 | 2 (8%) | 4 (24%) |  | 4 (15%) | 2 (12%) |  |
| Disease-free interval (d; MD; IQR) | 336 (0–762) | 634 (338–925) | 0.163 | 381.5 (33.5–869.5) | 558 (251–857) | 0.595 |
| Laterality of PM |  |  | 0.100 |  |  | 0.917 |
| Unilateral | 26 (90%) | 12 (71%) |  | 23 (82%) | 15 (83%) |  |
| Bilateral | 3 (10%) | 5 (29%) |  | 5 (18%) | 3 (17%) |  |
| Synchronicity |  |  | 0.258 |  |  | 0.311 |
| Synchronous | 2 (7%) | 3 (18%) |  | 2 (7%) | 3 (17%) |  |
| Metachronous | 27 (93%) | 14 (82%) |  | 26 (93%) | 15 (83%) |  |
| *BRAF* |  |  | >0.999 |  |  | >0.999 |
| Wild-type | 28 (97%) | 15 (94%) |  | 22 (96%) | 21 (95%) |  |
| Mutant | 1 (3%) | 1 (6%) |  | 1 (4%) | 1 (5%) |  |

RCSCS=Royal College of Surgeons Comorbidity Score. The chi-square test, Student’s T-test, and Mann-Whitney U-test were applied. **statistically significant at the level *p<*0.01. *statistically significant at the level *p*<0.05

**Table S10.** Associations of neoadjuvant therapy with M1- and M2-like macrophage densities and M1:M2 ratio in the first resected pulmonary metastases and primary tumours.

|  | Metastases | | | Primary tumours | | |
| --- | --- | --- | --- | --- | --- | --- |
|  | **Neoadjuvant chemotherapy** | | | **Neoadjuvant chemotherapy** | | |
|  | **No** | **Yes** |  | **No** | **Yes** |  |
|  | **MD (IQR)** | **MD (IQR)** | ***p*** | **MD (IQR)** | **MD (IQR)** | ***p*** |
| Tumour centre | | | | | | |
| Stromal macrophages | | | | | | |
| M1-like | 173.3 (96.1-312.4) | 145.9 (115.0-288.6) | 0.654 | 221.2 (116.6-383.6) | 282.6 (190.7-354.1) | 0.451 |
| M2-like | 247.6 (134.4-382.1) | 227.1 (85.4-377.3) | 0.350 | 116.2 (44.9-226.2) | 46.4 (16.6-159.8) | 0.254 |
| M1:M2 ratio | 0.7 (0.2-2.2) | 0.5 (0.4-2.3) | 0.958 | 2.2 (0.5-7.3) | 5.1 (1.6-26.8) | 0.181 |
| Epithelial macrophages | | | | | | |
| M1-like | 28.0 (13.8-115.8) | 40.2 (14.9-101.4) | 0.921 | 22.1 (9.1-64.8) | 41.2 (6.8-61.5) | 0.507 |
| M2-like | 18.7 (4.7-51.1) | 22.4 (4.3-40.1) | 0.676 | 3.5 (0.4-11.2) | 2.4 (0-12.6) | 0.746 |
| M1:M2 ratio | 2.6 (0.9-7.7) | 2.3 (0.9-13.7) | 0.480 | 6.4 (1-18.5) | 6.6 (3.8-684957779.2) | 0.219 |
| Invasive margin | | | | | | |
| Stromal macrophages | | | | | | |
| M1-like | 187.7 (121.4-229.2) | 197.3 (137.4-289.0) | 0.275 | 194.1 (133.8-482.1) | 227.5 (146.9-268.3) | 0.793 |
| M2-like | 310.5 (177.6-445.3) | 239.1 (162.9-398.5) | 0.064 | 166.3 (84.9-328.1) | 163.3 (4.1-215.7) | 0.261 |
| M1:M2 ratio | 0.5 (0.2-1.5) | 0.8 (0.4-1.7) | 0.061 | 1.2 (0.4-2.2) | 3.5 (0.6-45) | 0.249 |
| Epithelial macrophages | | | | | | |
| M1-like | 104.0 (52.3-182.8) | 60.8 (22.4-144.9) | 0.627 | 17 (4.8-66) | 95.4 (62.8-114.7) | **0.016*** |
| M2-like | 45.4 (20.3-135.5) | 29.9 (7.6-57.5) | 0.481 | 6 (0-15.4) | 1.3 (0-81.9) | 0.750 |
| M1:M2 ratio | 1.0 (0.5-3.1) | 2.8 (1.3-5.1) | 0.298 | 4.2 (0.6-247269587.4) | 543824134.5 (2.2-8843607435) | 0.075 |

*statistically significant at the level *p*<0.05

**
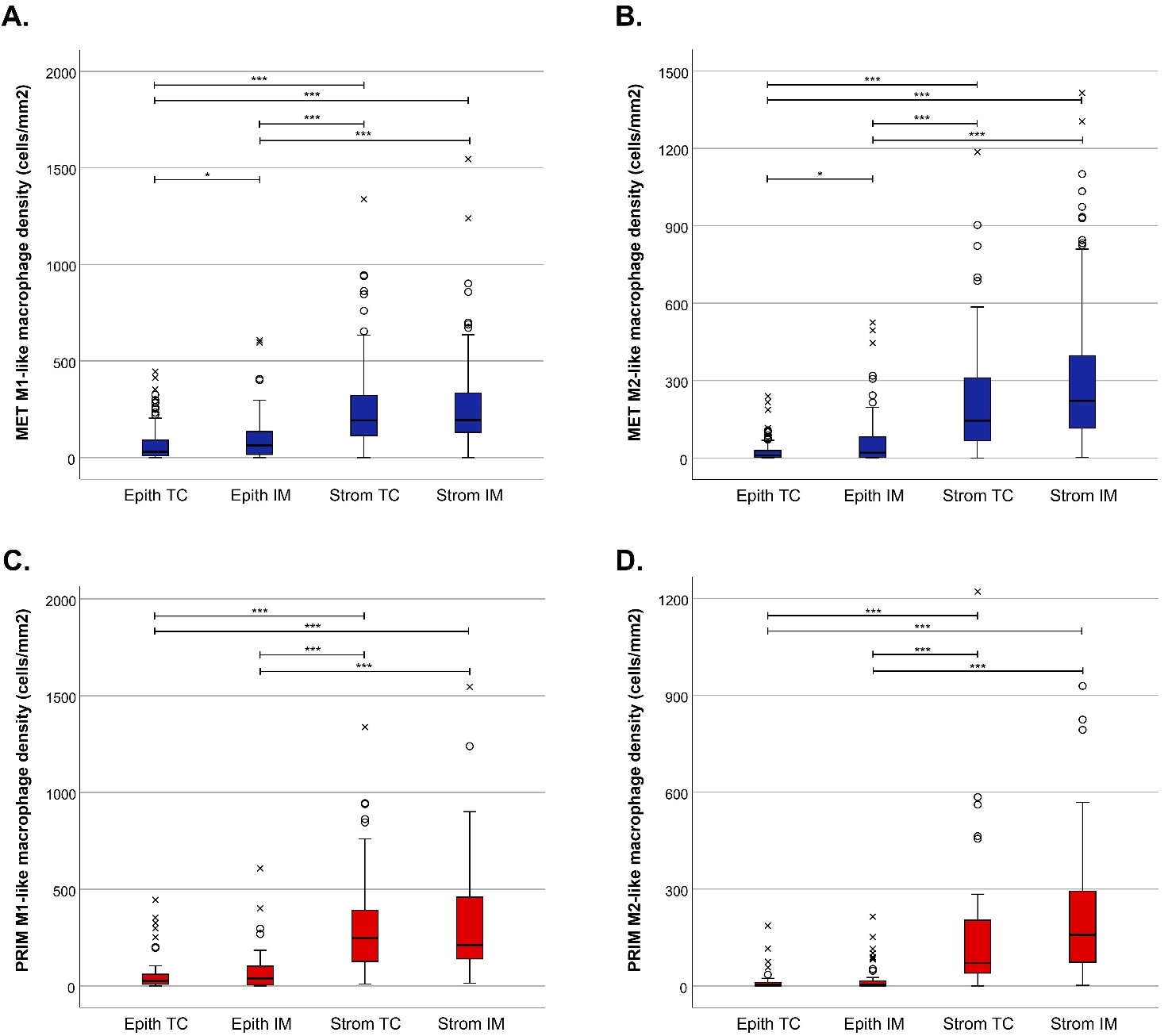
**

**Figure S1**. Comparison of M1- and M2-like macrophage densities in the different compartments of the first resected pulmonary metastases (**A** and **B**) and primary CRC tumours (**C** and **D**). Kruskal-Wallis tests were applied. Dunn’s multiple comparison tests were used for post-hoc analysis. *** Significant at the level *p*<0.001. * Significant at the level *p*<0.05. Circles indicate outliers and crosses extreme outliers. Epith=epithelial; IM=invasive margin; Strom=stromal; TC=tumour centre.

**
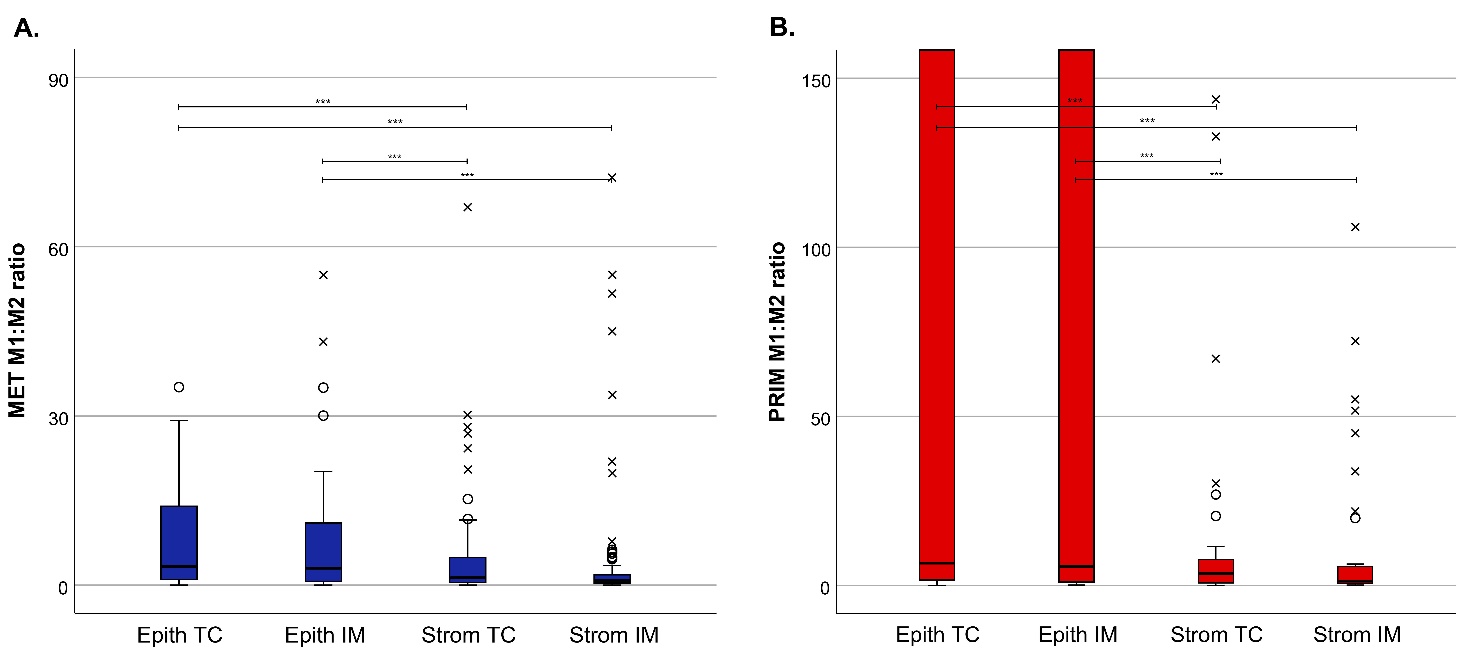
**

**Figure S2.** Comparison of M1:M2 ratios in the different areas of the first resected pulmonary metastases (**A**) and primary CRC tumours (**B**). Kruskal-Wallis tests were applied. Dunn’s multiple comparison tests were used for post-hoc analysis. *** Significant at the level *p*<0.001. Circles indicate outliers and crosses extreme outliers. Epith=epithelial; IM=invasive margin; Strom=stromal; TC=tumour centre.


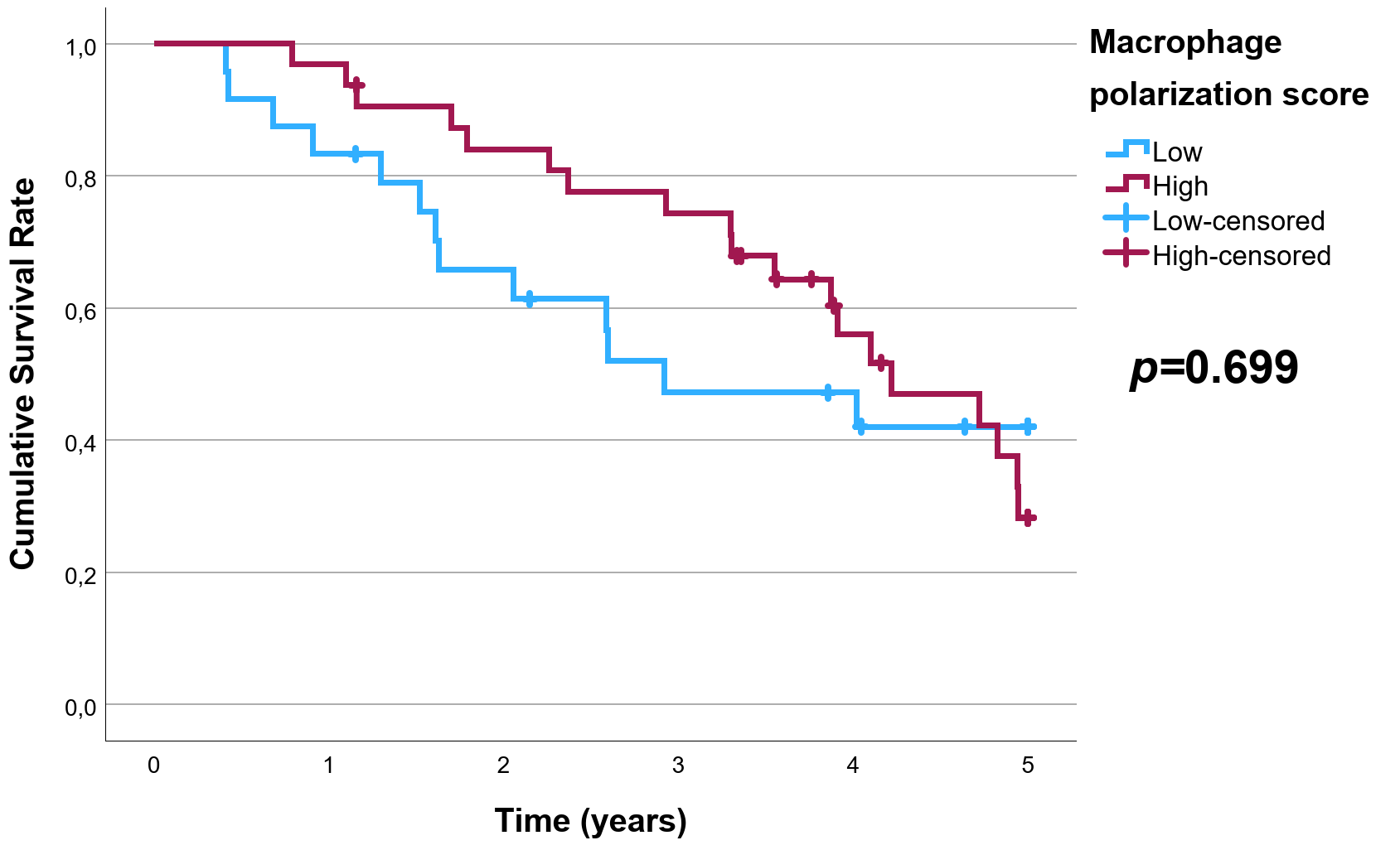


**Figure S3.** Kaplan-Meier survival analysis of an integrative macrophage polarization score using the density ratios of all M1-like and M2-like macrophages within each resected pulmonary metastasis (including the intraepithelial and stromal areas of both invasive margin and centre of the tumour). Log rank tests were applied.


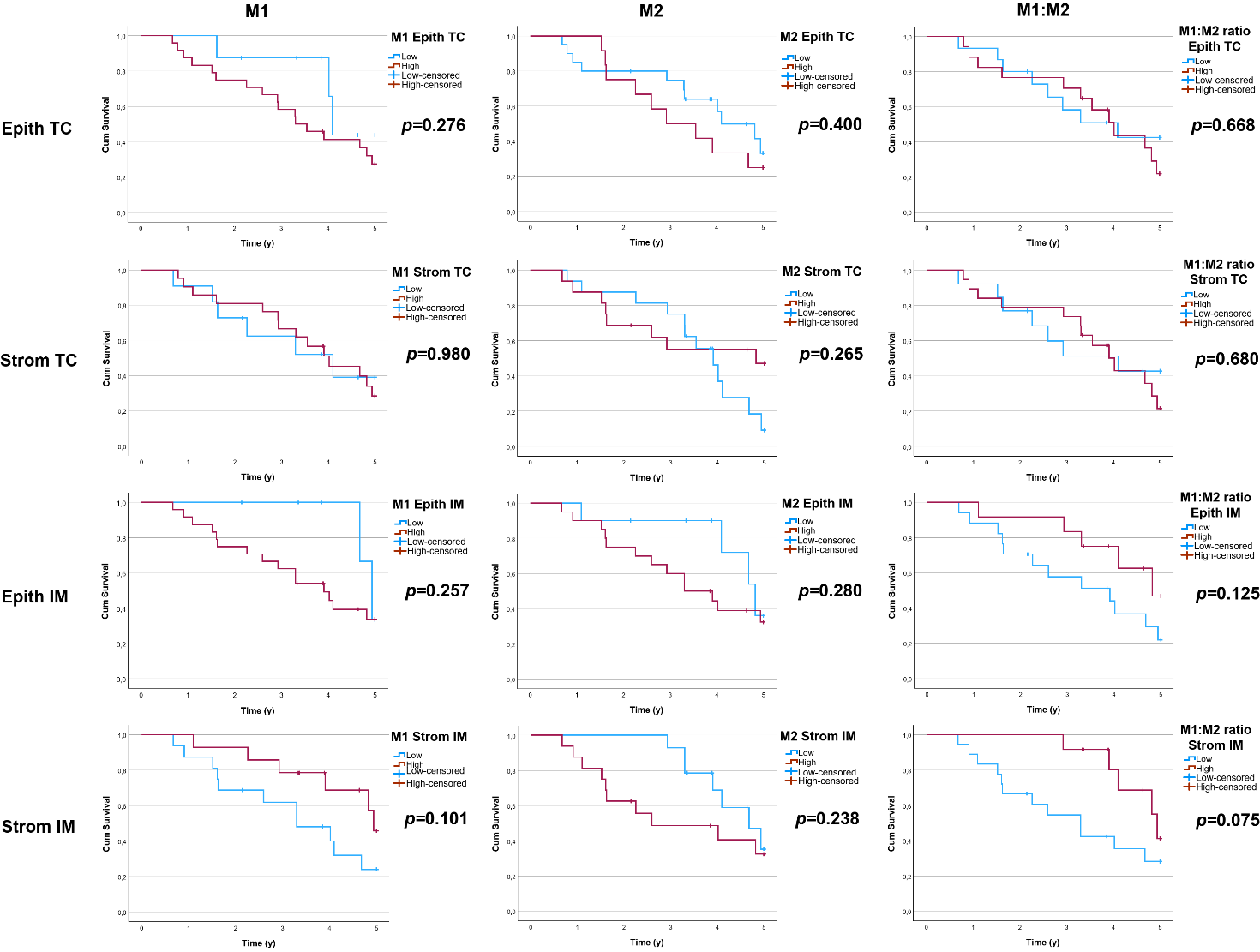


**Figure S4.** Kaplan Meier curves of 5-year overall survival after pulmonary metastasectomy stratified by M1-like, and M2-like macrophage densities and M1:M2 density ratios in the epithelial (Epith) and stromal (Strom) compartments of the invasive margin (IM) and tumour centre (TC) of the first resected pulmonary metastases in patients not receiving neoadjuvant therapy. Log rank tests were applied.
